# Traumatic rupture of the posterior deltoid tendon during weight lifting: A case report and review of literature

**DOI:** 10.1002/ccr3.3710

**Published:** 2021-04-09

**Authors:** Brent Sanderson, Michael Bogard, Reza Jazayeri

**Affiliations:** ^1^ Community Memorial Hospital Ventura CA USA; ^2^ Kaiser Permanente of Southern California Los Angeles CA USA

**Keywords:** Deltoid, Suspensory fixation, Weightlifting

## Abstract

Deltoid tendon humeral sided avulsion leads to discomfort and functional limitation in the young active population. This report illustrates a case for surgical treatment with a simple suspensory device that allows for early return to activity.

## Graphical abstract

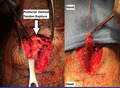

## 1 INTRODUCTION

Deltoid tendon rupture is an infrequent injury. We report on a young active white man who sustained a humeral sided deltoid avulsion tendon rupture while attempting a 450‐pound barbell shoulder shrug. Early surgical fixation and a progressive rehabilitation program provided successful short‐ and long‐term outcomes in our young active patient.

Rupture of the deltoid is reported most frequently in elderly patients with associated massive rotator cuff tears.^1^, ^2^ Other reported deltoid tears have been documented following open surgical procedures that violate the deltoid muscle fibers.^3^ Only a handful of cases have been recorded that involve young active patients who suffered traumatic deltoid tendon ruptures.^4^, ^5^, ^6^ The deltoid originates from the anterior border of the clavicle, acromion, and spine of the scapula, inserting distally at the deltoid tuberosity. Detachment of the deltoid is associated with an acute, sudden onset of shoulder weakness and pain. All of the literature to date describes deltoid detachment from its origin on either the acromion or the clavicle.^1^, ^2^, ^3^, ^4^, ^5^, ^6^ There is a paucity of cases described in the literature involving traumatic deltoid ruptures from the humeral insertion site. With the aid of a cadaveric and muscle activation study, we postulate a mechanism of injury and discuss our intraoperative findings.^7^, ^8^ The aim of our study was to summarize the current literature on deltoid ruptures and present a case report and surgical technique.

## 2 CASE REPORT

An active 37‐year‐old, right‐hand dominant man presented to the clinic for left shoulder pain and weakness that began 7 days prior while lifting weights. His symptoms started abruptly after completion of a set of barbell shoulder shrugs with 450 pounds. During the action, he felt a popping sensation over the left posterior shoulder, followed by sharp pain. There was associated deformity and swelling following the injury. He was employed as a heavy laborer and felt his work was limited by these new symptoms. He denied any previous shoulder trauma, injury, or prior shoulder girdle corticosteroid injections. On physical examination, there was evident point tenderness over the insertion site of posterior deltoid fibers on the humerus and in the main deltoid muscle belly. During strength testing of the left shoulder abduction, the posterior deltoid was graded 4/5.

**FIGURE 1:**
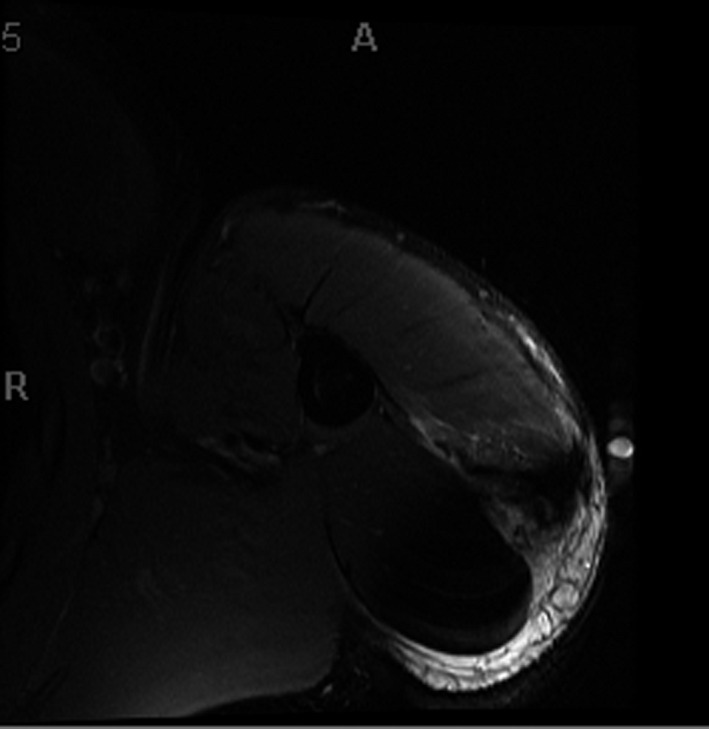
Left shoulder MRI axial STIR image demonstrating a posterior head of the deltoid muscle‐tendon avulsion from the deltoid tuberosity of the humerus. There is approximately 3 cm retraction of the torn fibers. There is associated muscle strain of the posterior head of the deltoid with hematoma formation

Radiographs of the patient's shoulder showed no fracture, subluxation, dislocation, abnormal calcifications, or narrowing of the acromiohumeral interval. MRI revealed tendon avulsion of the posterior head of the deltoid muscle from the deltoid tuberosity of the humerus (Figure 1).

## 3 SURGICAL FIXATION

Following standard preoperative consent and induction of general anesthesia, he was placed in the lateral decubitus position, and the left upper extremity was prepped and draped in the usual sterile fashion.

**FIGURE 2:**
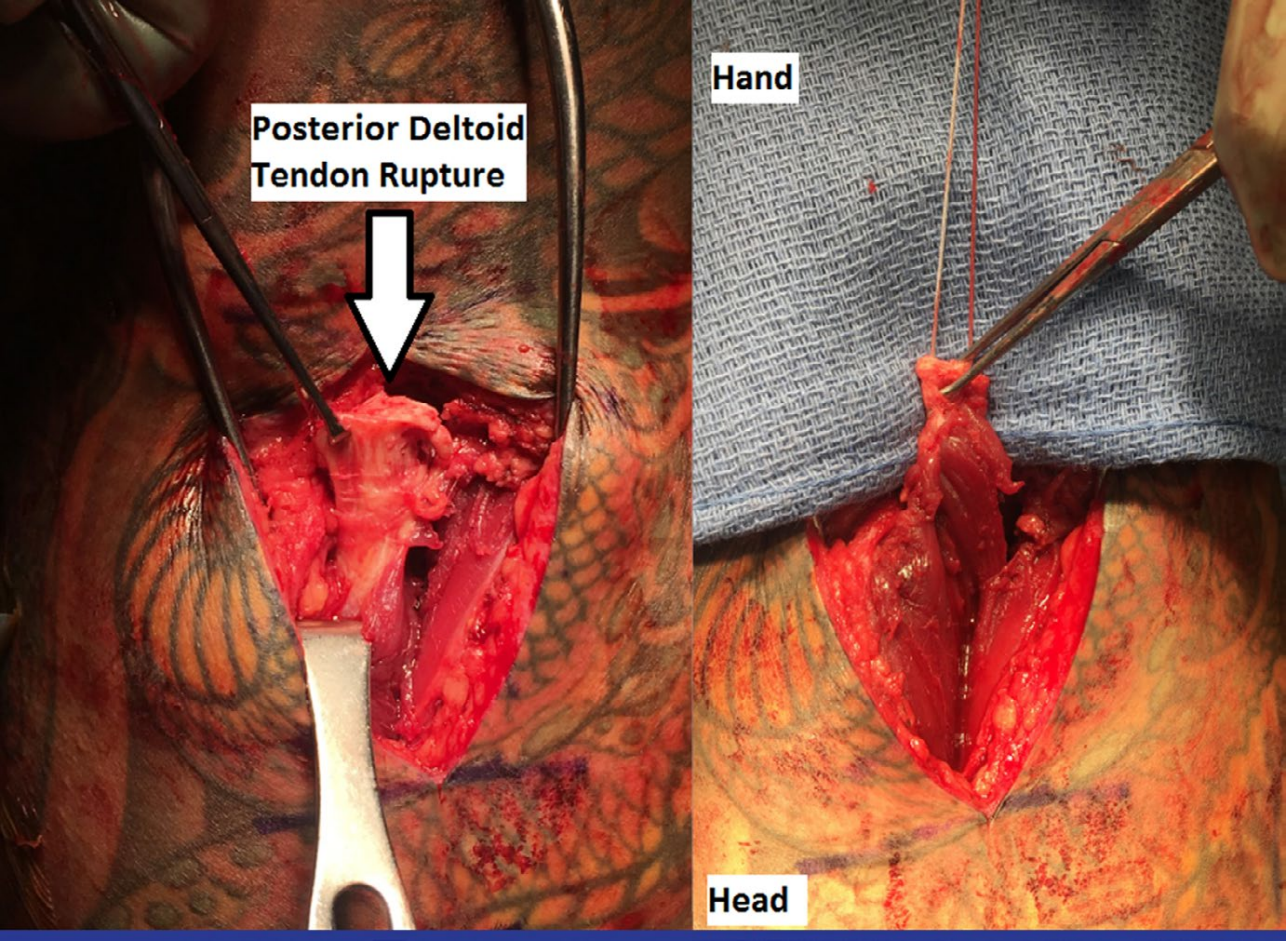
Left shoulder clinical images demonstrating the posterior deltoid tendon rupture (left) and suture fixation (right) prior to cortical button fixation to the humeral insertion site

A 7 cm longitudinal lateral shoulder incision was then made over the deltoid insertion on the humerus. The distal posterior deltoid tendon end was identified, and a nonabsorbable high‐strength suture loop (FiberWire No. 2; Arthrex) was utilized to place a four‐throw whipstitch through the distal tendon (Figure 2).

Careful dissection was performed down to the deltoid tuberosity along the tract left from the avulsed posterior tendon. A pilot hole was drilled across near cortex of the humeral shaft. This was performed at the anatomic insertion point of the tuberosity. The suture ends were next passed through the cortical button (TightRope; Arthrex). The cortical button was inserted and flipped on the near cortex of the humerus. The sutures were pulled, bringing the tendon securely down to the tuberosity of the humerus. A free needle was used to suture to the proximal aspect of the tendon with a Krackow suture technique. After three locking throws each, the two suture ends were then tied approximately 1.5 cm from the tuberosity. The wound was then copiously irrigated and closed in the standard fashion. The patient's operative shoulder was placed into a sling immobilizer.

The patient started a progressive supervised rehabilitation program beginning with passive shoulder elevation and pendulum exercises starting 1 week after surgery. Three weeks following surgery, supervised active shoulder range of motion was allowed. This was followed by shoulder girdle strengthening 8 weeks postoperatively. Focused strengthening of the shoulder started with forward flexion, and internal and external rotation. Shoulder abduction strengthening was then pursued following adequate range of motion and forward flexion strength. Our patient was able to return to work full‐time and pain‐free 4 months postoperatively. At the 6‐month postoperative visit, the patient's left shoulder returned to preinjury level. Range of motion of the operative shoulder was 30 degree of extension to 170 degrees of flexion and abduction. Strength of the deltoid was measured to be 5/5 on physical examination. At 2 years following the surgical procedure, the patient had returned to all weight lifting with no limitations, pain, or complaints (Video S1).

## 4 DISCUSSION

Current literature review demonstrates no case reports describing documenting distal deltoid tendon rupture from the deltoid tuberosity. This case vignette is the first to describe such injury of humeral site deltoid tendon rupture and describes successful surgical repair. In contrast to our distal deltoid tear, there have been multiple reports on proximal deltoid origin site detachment from the acromion and distal clavicle.^4^, ^5^, ^6^ The mechanism of injury described by prior research for this rare injury includes seatbelt‐induced injury after a motor vehicle accident, cricket bowling maneuver, and pull‐up exercises.^4^, ^5^, ^6^

Our patient was performing barbell shoulder shrugs with 450 pounds when he experienced acute posterior left shoulder pain during the eccentric phase of the exercise. Andersen and colleagues evaluated the shoulder girdle muscle activity using surface electromyography in a cohort of middle‐aged females.^7^ The level of muscle activation was measured as a percentage of maximal voluntary static contraction. Activation of the posterior portion of the deltoid muscle was found to be significantly higher during exercises that involved a forward incline of the body including reverse flys (102 ± 9%), one hand rows (83 ± 6%), and shoulder shrugs (71 ± 5%). Comparing this to our study participant, he described a slight forward flexion at his waist in order to bring the barbell out from rubbing on his thighs during the shrug exercise. This suggests an increase forced vector directed through the posterior deltoid fibers, which may have contributed to the tendon rupture. There are some limitations of direct comparison between our patient and the reported data. The shrugs exercise performed by Andersen's female patients utilized dumbbells to the side rather than a barbell.

A recent study analyzing the anatomy of the deltoid muscle tendons examined eight cadaver specimens with an average age of 76 years identifying the deltoid origins and end tendons insertion sites. The final model from the study consisted of an end tendon consisting of a continuous succession of bipennate end tendon blades centrally interspaced by unipennate tendon parts, creating a natural segmentation. They identified an average of 2.9 ± 0.8 end tendon blades inserting posteriorly at the lateral humerus.^8^ This is consistent with our intraoperative findings and successful fixation of two bipennate posterior end tendons. There is currently no research on the clinical significance of individual end tendon ruptures.

Treatment of isolated proximal deltoid tears has generated controversy due to limited data on the subject and the diversity of patients currently reported on. Support for surgical fixation in healthy active individuals based on case report data demonstrated satisfactory results following operative treatment.^5^, ^6^, ^7^ Following operative fixation, patients regained functional deltoid strength and experienced painless range of motion after completing postoperative physical therapy. Support for nonoperative therapy with a supervised rehabilitation program stems from a case report on a partial deltoid detachment from the posterolateral acromion in a healthy 31‐year‐old cricket player. Nonoperative treatment including indomethacin treatment and physical therapy resulted in a return of function in 8 weeks. However, the patient's MRI was complicated by areas of myositis ossificans and heterotopic calcification present at the posterolateral acromion.^4^

This case is unique due to the posterior deltoid tendon rupture, with confirmed tendon retraction on magnetic resonance imaging; additionally, the patient had physical deformity with associated weakness and pain affecting his occupation. Operative management was successful and allowed him to return to work with similar preinjury strength, range of motion, and functional capacity within 4 months of surgery.

## CONFLICT OF INTEREST

The authors, their immediate families, and any research foundations with which they are affiliated did not receive any financial payments or other benefits from any commercial entity related to the subject of this article.

## AUTHOR CONTRIBUTIONS

BS: developed the research plan and concept, and wrote and edited the manuscript. MB: acquired data, and wrote and edited the manuscript. RJ: revised the manuscript critically and provided suggestions for final preparation of the manuscript.

## ETHICAL APPROVAL

The patient consented to the publication of his case.

## Supporting information

Video S1

## Data Availability

All data regarding the above case are present within this manuscript.
